# Predictability in evolution: Adaptation of the Bonaire anole (*Anolis bonairensis*) to an extreme environment

**DOI:** 10.1371/journal.pone.0176434

**Published:** 2017-05-01

**Authors:** Roger S. Thorpe

**Affiliations:** School of Biological Sciences, Bangor University, Bangor, United Kingdom; Fred Hutchinson Cancer Research Center, UNITED STATES

## Abstract

The extent to which evolution is deterministic (predictable), or random, is a fundamental question in evolution. This case study attempts to determine the extent to which interspecific divergence can be predicted from intraspecific trends related species. The mountainous Lesser Antilles are occupied by one or two anole species with very substantial intraspecific differences in the quantitative traits between xeric and rainforest habitats. These ecologically determined differences tend to be in parallel in each island species. A related species (*Anolis bonairensis*) lives on the far more xeric island of Bonaire, and this study tests the extent to which its interspecific divergence in hue and pattern traits can be predicted from the parallel intraspecific variation exhibited in Lesser Antillean anoles. Regression against a multivariate climate variable suggests that the hue and pattern of the Bonaire anole are consistently predicted from the ecologically determined intraspecific variation of its Lesser Antillean relatives. However, this predictability may be less consistent with other character systems, for example, scalation.

## Introduction

Evolutionary studies may emphasise chance, or unrepeatable contingency, as in Gould’s [1] classic study of the Burgess Shales, and this approach can find its extreme expression in attempts to apply chaos theory to evolution [2]. On the other hand studies may focus on repeatability, or determinism, in evolution [3–5] as this can enable workers to elucidate the factors which that are impacting on the evolutionary process. Simple convergence of an unrelated pair of species such green tree pythons and green tree boas [6] exemplify this, as does mimicry of unrelated forms [7], and the more complex convergence in community structure exemplified by Greater Antillean anoles [8]. Central to the “chance versus determinism” debate is the extent to which intraspecific microevolutionary trends can predict interspecific macroevolutionary trends. An approach centred on punctuated equilibria [1,2,9] would minimise this predictability, while an approach centred on natural selection driven evolution would not.

The younger, mountainous Lesser Antilles (LA) have pronounced climatic differences between montane rainforest and xeric, coastal, rain-shadow, habitats, and climatic differences among islands are trivial compared to these within-island differences [10]. These islands are all occupied by solitary anoles (or at most two endemic species per island) from the northern *bimaculatus* series, or southern *roquet* series. Parallel intraspecific variation between xeric and rainforest habitats is widespread in these Lesser Antillean anoles across a range of traits [10], suggesting adaptation by natural selection to these different habitats. To what extent can this intraspecific parallel evolution predict interspecific evolutionary differences?

The relative similarity of the climate regimes on the montane Lesser Antilles does not allow such a test. However, the island of Bonaire, north of the South American coast, is occupied by a member of the otherwise southern Lesser Antillean *roquet* series anoles (*Anolis bonairensis*), and this relatively low elevation island (240m in Bonaire compared to 1397m, 1447m, and 1467m in Martinique, Dominica and Basse Terre respectively) is extremely xeric relative to the Lesser Antilles. For example, using Worldclim data and the site employed in this study and Thorpe et al [10], annual precipitation is from 165, 150, and 154 cm on the xeric coasts of Basse Terre, Dominica and Martinique respectively) to 344, 378 and 394cm in their rainforest sites. Whereas, annual rainfall is much lower and varies little among sites in Bonaire (43-51cm). This paper attempts to assess the extent to which the parallel intraspecific xeric v rainforest variation in Lesser Antillean anoles can predict the phenotype of the Bonaire anoles across a range of traits from hue, pattern and scalation, and hence, contribute to the debate concerning the extent to which microevolutionary trends can predict interspecific evolution.

Across the Lesser Antillean anoles as a whole, the robust generalization is that although there may be pronounced phylogeographic lineages within an island species, the quantitative traits (QTs) of an anole primarily reflect the habitat differences within an island and not the phylogeographic lineages. Nevertheless, this study takes a conservative approach and attempts to establish to what extent the Bonaire anoles have distinct phylogeographic lineages and establish appropriate sampling to test the predictability of the evolution of given quantitative traits.

## Materials and methods

### Phylogeography of *A*. *bonairensis*

**Fig 1:**
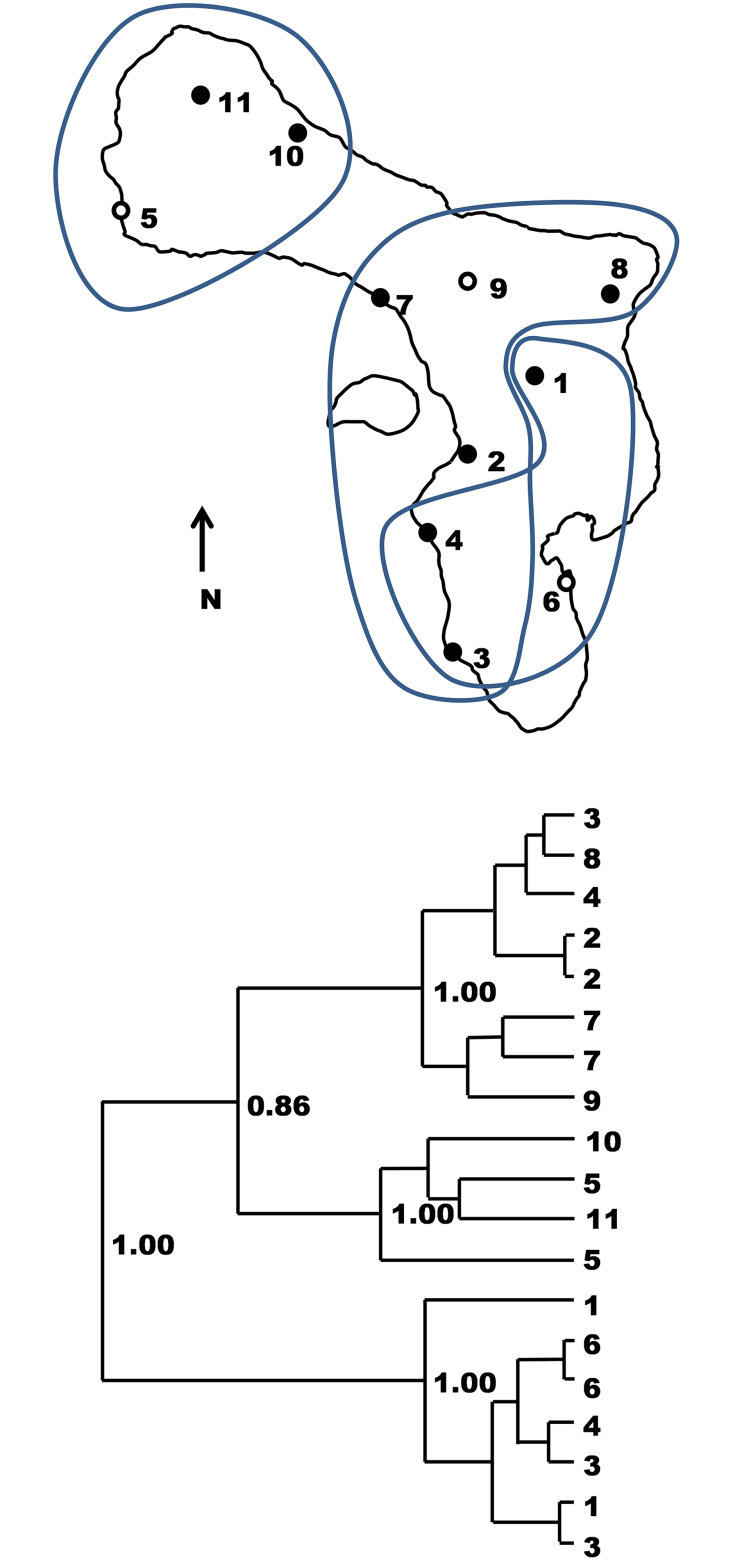
Bonaire site localities and phylogeography. Individuals from localities 1–9 are used in the phylogeography (terminal nodes of tree and circles on map) and quantitative traits are studies from localities 5, 6 and 9 (white centred circles). The MT-CYB gene tree (posterior probabilities for key nodes) shows three main lineages, their partly overlapping distributions being ringed in light grey on the map.

The evidence accumulated across a diverse range of comprehensive studies of Lesser Anillean anoles suggests that phylogeographc relations, albeit pronounced in some cases, have little impact on the geographic variation of QTs within a species compared to current climatic conditions ([10 –12] and references therein). These studies include field experiments on natural selection and common garden experiments [12–13], extensive studies showing that QTs primarily correlate with habitat and climate, but not phylogeography ([10 –18] and references therein), studies of rapid adaptation in an invasive species [19], and studies of parallel evolution [10–11]. Nevertheless, this study evaluates the phylogeographic relationships in *A*. *bonairensis* to inform the selection of study sites. Phylogeographic relationships for *Anolis bonairensis* were evaluated over all eleven sites (Fig 1). Specimens were captured by hand, and tissue for a DNA sample obtained by inducing natural autotomy of about one centimetre of tail tip. The specimens were released unharmed at the point of capture. These procedures (and those employed for QT recording below) were approved by the College of Natural Sciences ethics committee of Bangor University, and DROB, Section of Environment and Natural Resources, Bonaire).

Novel DNA sequences from Bonaire (GenBank accession numbers: KY964635- KY964653) were assembled with those from Lesser Antillean *Anolis* outgroup, using published sequences ([10] and references therein). The mitochondrial cytochrome b gene (MT-CYB) was used as a marker for all species, the Lesser Antillean sequences being the effective outgroup. This marker has provided a high level of variability and resolution between phylogeographic lineages in this genus [14, 20]. Mitochondrial fragments for MT-CYB were PCR-amplified using the primers MTA-S (5′-ATCTCAGCATGATGAAACTTCG-3′) and MTF-S (5′-TTTGGTTTACAAGACCAATG-3′) as in [21].

Relationships and divergence time among haplotypes were estimated using a Bayesian approach in BEAST v. 1.8.2. [22]. The tree was calibrated by constraining the age of the tree root, based on reanalysis of the data and calibrations used in a previous phylogenetic study of iguanian lizards [23] which indicated a mean age of the *Anolis* crown group of 44.9my, with a 95% HPD of 36.1–53.3my (T. Townsend, pers. Comm.). Accordingly, in our analysis, an informative normal prior was placed on the age of the tree root with a mean age of 44.9my and standard deviation of 4.5my. The mean per-lineage substitution rate was estimated within an uninformative uniform prior of 0–2% per million years. A Yule process was used as the tree prior, and the HKY+I+G was selected as an appropriate nucleotide substitution model, under the Bayesian Information Criterion in MEGA 5 [24]. Preliminary runs using a lognormal relaxed cock model failed to reject zero variation in substitution rate across the tree, and so a strict clock model was utilised. MCMC chains ran for sufficient length to achieve convergence and sufficient sampling of all parameters (ESS > 200), verified using the program TRACER v. 1.6 [25]. The maximum clade-credibility (MCC) tree was obtained with node heights scaled to the median of the posterior sample using the program TreeAnnotator.

### Climate

The climate data for all sites across Bonaire and the Lesser Antilles [10] were downloaded at 30 arc-second resolution from the WorldClim website (www.worldclim.org). Principal components (PCs) were extracted from the variance—covariance matrix where each site (irrespective of island) is a case and the 19 log_e_-transformed climate traits are variables. The first PC optimizes the ordination of climate differences among sites. The details are in [10], and the data and PC scores for the Lesser Antillean and Bonaire sites selected for this study are in S1 File.

### Quantitative traits

For A. *bonairensis*, subsequent to the phylogeographic analysis, quantitative traits were recorded from high-quality digital macrophotographs taken from 22 live male specimens across three sites (S2 File), with the specimens being released at point of capture. Localities 5 Nukova (northwest), 6 Sorobon (southeast) and 9 Boka Olivia (central) were used to represent the geographic regions of Bonaire and phylogeographic lineages (Fig 1). Preliminary (and final) results indicated that, relative to the intraspecific comparisons within Lesser Antillean anoles, the QTs and climate within Bonaire vary little and these localities are likely to be representative.

Where complete, well supported phylogenies are not available across the entire sampled taxon range, selected comparisons within a lineage are appropriate for controlling for phylogeny [26]. This is the case with this data set where aspects of the interspecific relationships have yet to be well resolved, some species do not have a well-resolved phylogeographic structure, and/or, phylogeographic divisions may overlap geographically [14]. Even though the overwhelming evidence is that QTs are not primarily determined by intraspecific phylogeny (see above), this study controls for phylogeny by selected sites from just one primary phylogeographic area. Hence, for a given species, replicate sites are selected from both xeric and rainforest habitats in accordance with the phylogeography of the individual species. See Thorpe et al [10] for the details of the sampling of the Lesser Antillean anoles.

For the *bimaculatus* series this his gave 2 xeric and 2 rainforest sites for *A*. *marmoratus* (Basse Terre), and 9 xeric and 6 rainforest sites for *A*. *oculatus* (Dominica). For the *roquet* series this gave 4 xeric and 4 rainforest sites for *A*. *roquet* (NW Martinique), 2 xeric and 2 rainforest sites for *A*. *roquet* (central Martinique), 4 xeric and 6 rainforest sites for *A*. *luciae* (St Lucia), 2 xeric and 2 rainforest sites for *A*. *trinitatis* (St Vincent), *A*. *aeneus* (Grenada) and *A*. *richardii* (Grenada).

The quantitative traits tested are those that showed significant pervasive parallel evolution in one or both anole series (*bimaculatus* and *roquet*) from the Lesser Antilles [10], and that could be readily compared among different recorders (islands). This gave three hue traits (dorsal green, dorsal blue, and achromatic dorsal), one pattern trait (chevron intensity), and two scalation traits (dorsal scales along trunk length, ventral scales along trunk length). See Thorpe et al [10] for trait definitions, and S2 File for QT data.

To test the prediction that the minimal variation in climate across Bonaire would result in little or no geographic (among site) variation in QTs one way ANOVAs were run with the traits as variables and the sites as groups. Whether, or not, the state of a QT can be predicted in the extreme xeric habitat of Bonaire was investigated by plotting the trait (dependent variable) against the principal component representing climate (independent variable) for all sites for all included species. Only species with recorded comparable traits and significant difference between xeric and rainforest sites were included [10]. Trait means at each site were employed. A regression line and correlation are computed (excluding the Bonaire sites), and the prediction interval curves fitted to the plot (IBM Corp. Released 2013. IBM SPSS Statistics for Windows, Version 22.0. Armonk, NY: IBM Corp.). These curves give the 95% chance of a data point on y being within these limits at a given value of x (NB they are not the confidence limits of the slope). For the state of a trait in Bonaire to be correctly predicted the correlation/regression must be significant and the site means must fall between the upper and lower prediction intervals. Although this model allows for the computation of prediction interval curves, it combines some interspecific variance with the dominant intraspecific variance. However, the pooled within-species regression slope is very close to the employed regression slope and, for each trait, their 95% confidence limits overlap (S1 Table).

## Results

### Phylogeography of *A*. *bonairensis*.

The Bonaire anole appears to be around 1.21my (95% HPD 0.73–1.78my) old judging from deepest intraspecific node. Three phylogenetic groups can be recognised (Fig 1) with some geographic overlap, a south/southeastern group (the most basal and including sites 1,3,4,6), which partly overlaps with a central/southwestern group (sites 2, 3, 4, 7, 8, 9), and a northern group (sites 5, 10, 11). Sites for which QTs are studied (5, 6, 9) represent all three lineages.

### Climate

The major principal component ordinates between xeric and rainforest sites (xeric low, rainforest high, values), with the three Bonaire sites all having similar, and extremely xeric climates. Bonaire’s climate is far more xeric than even the most xeric sites in the high elevation Lesser Antillean islands studied (S1 File).

### Quantitative traits

**Fig 2:**
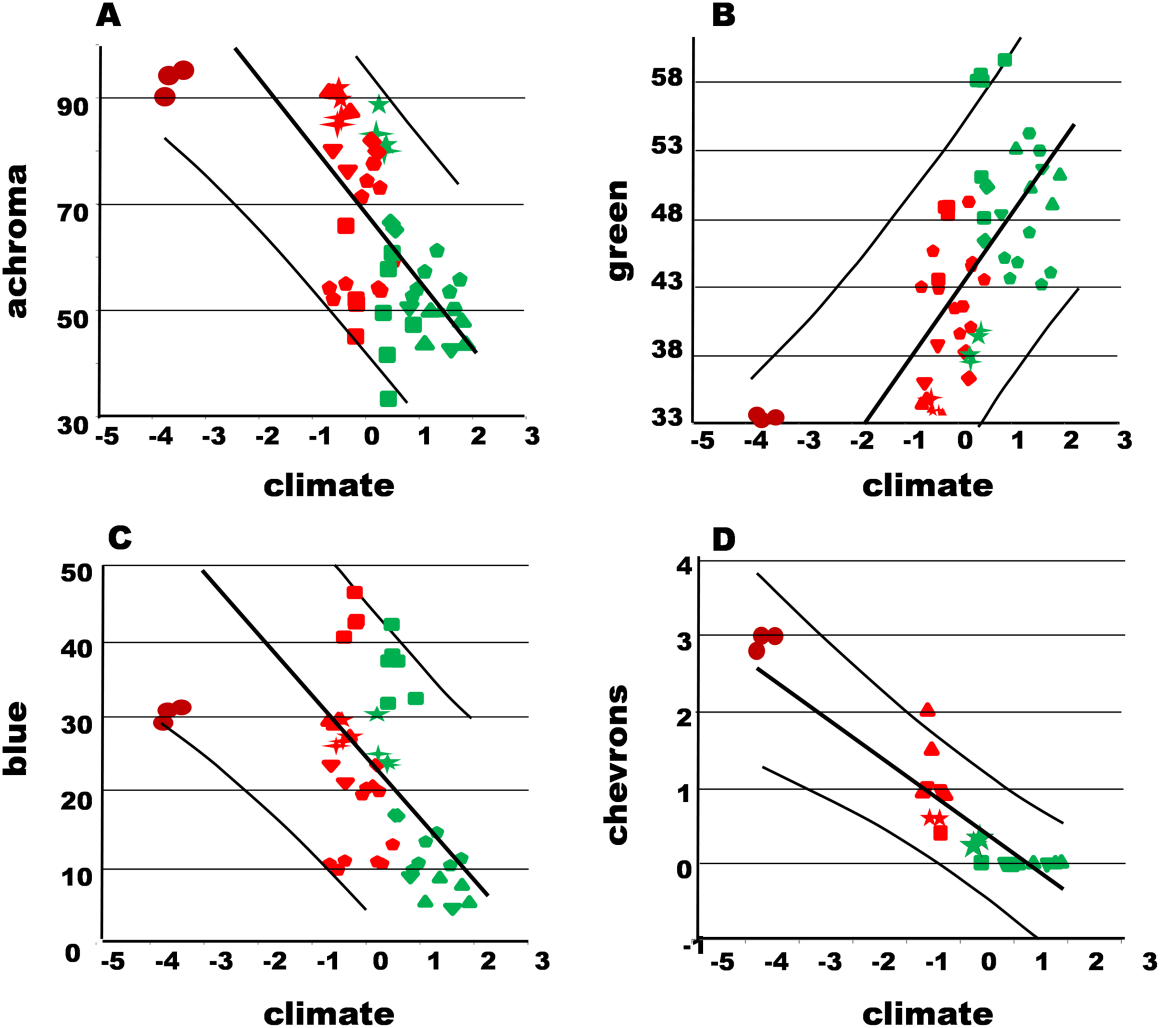
Regression plots for individual traits against climate. Horizontal axis pc1representing climate, with xeric low scores and montane rainforest high scores. A achromatic dorsum, B greenness of dorsum, C blueness of dorsum, and D intensity of dorsal chevrons. Regression slope (straight line) with upper and lower prediction interval curves for scatter points. Species symbols (site means, xeric red, montane green), *A*. *marmoratus* Basse Terre hexagonal, *A*. *oculatus* Dominica pentangle, *A*. *roquet* NW Martinique upright triangle, *A*. *roquet* Central Martinique inverted triangle, *A*. *luciae* St Lucia square, *A*. *trinitatis* St Vincent diamond, *A*. *aeneus* Grenada five point star, *A*. *richardii* Grenada four point star, and *A*. *bonairensis* Bonaire circle.

The Bonaire sites all had similar values for the traits (Fig 2A–2D, S3 File), there being very little geographic variation within *A*. *bonairensis*. When tested by ANOVAs, VLS was only marginally significant (F = 3.6 P = 0.05), and none of the remaining QTs had significant differences among sites (achromatic F = 2.9 P > 0.05, green F = 0.7 P > 0.05, blue F = 2.8 P >0.05, dorsals F = 0.7 P> 0.05) (S2 Table). When Bonferroni tested, no traits were significant. Chevron intensity was untested as it showed too little variance within and between Bonaire sites.

The hue and pattern traits (achroma, green, and blue dorsum, and chevron intensity) were comparable across sufficient species given the above criteria (8, 8, 7, 4 species respectively), but the ventral and dorsal scalation were not. Consequently, these scalation traits were not analysed further (see S3 File for further information on scalation).

There is a significant negative correlation/regression (Table 1) between achromatic dorsal (greyness) and climate in LA anoles. The Bonaire anole is very achromatic (S1 Fig) as predicted by the situation in xeric LA anoles, with the means falling between the prediction intervals (Fig 2A). There is a significant positive correlation/regression between dorsal greenness and climate in LA anoles (Table 1). The Bonaire anole lacks green coloration as predicted by the situation in xeric LA anoles, with the means falling between the prediction intervals (Fig 2B). There is a significant negative correlation/regression (Table 1) between dorsal blueness in Lesser Antillean anoles. The Bonaire anole is has fairly high levels of blueness as predicted by the situation in xeric LA anoles, with the means falling between the prediction intervals (Fig 2C). There is a significant negative correlation/regression (Table 1) between chevron intensity and climate in LA anoles. The Bonaire anole has very pronounced chevrons as predicted by the situation in xeric LA anoles, with the means falling between the prediction intervals (Fig 1D).

**Table 1:** Regression statistics for quantitative traits.

Trait	N^a^	r^b^	p^c^	a^d^	b^e^
Achromatic dorsum^1^	53	-0.60	<0.001	68.56	-13.15
Green dorsum^1^	53	0.58	<0.001	42.40	5.64
Blue dorsum^2^	49	-0.52	<0.001	24.39	-8.15
Chevron intensity^3^	19	-0.78	<0.001	0.66	-0.51

^**a**^ Sample size.

^**b**^ Correlation of QT and climate.

^**c, d, e**^ null hypothesis probability, intercept and slope of regression of QT against climate.

^1^ Site means from all studied species.

^2^ Site means from all studied species included except *A*. *marmoratus* from Basse Terre.

^3^ Site means from *A*. *lucia* (St Lucia), *A*.*aeneus* (Grenada) and *A*.*roquet* from northwest and central Martinique.

## Discussion

Unlike LA anoles from mountainous islands, the Bonaire anole shows little or no geographic variation in the QTs studied. The phylogeographic analysis revealed the within-island (a conservative estimate) divergence to be 1.2mybp. This is a conservative estimate, compared to between-species divergence, as incomplete sampling and haplotype loss will under-estimate divergence time. Natural selection experiments [8, 12, 13] and divergence of recent colonizers [19] indicate that the quantitative traits of anoles can diverge very rapidly when subjected to directional selection. Consequently, the divergence time and phylogeographic structure indicate that Bonaire has been occupied by this anole long enough for quantitative traits to diverge if subject to directional selection, and any lack of geographic variation in the Bonaire anole cannot be attributed to very recent colonization. In contrast, the substantial intraspecific variation of QTs in anoles from mountainous Lesser Antillean islands is primarily associated with substantial altitudinal climate and associated habitat differences [10–18]. This predicts that the lack of substantial altitudinal variation within Bonaire and the consequent relative uniformity of habitat and climate, should result in little, or no, geographic variation in quantitative traits in *A*. *bonairensis*. This generalization extends to other regions and lizard taxa, such as Canary island lacertids [27], skinks [28], and geckos [29] where geographic variation of traits within an island species is primarily associated with climatic/habitat differences. Moreover, as is largely the case in Lesser Antillean anoles, there may be distinct intraspecific lineages in the Bonaire anole, but these are not associated with divergence in quantitative traits.

The Bonaire anole, like the anoles from the Lesser Antilles from xeric regions, is largely achromatic, with comparatively low levels of green and medium levels of blue (S1 Fig). In those Lesser Antillean species with the capacity for chevrons, the xeric forms have well developed chevrons and predictably the Bonaire anole has very intense chevrons. The visual appearance of the Bonaire anole (S1 Fig) is therefore very much as predicted from the xeric Lesser Antillean forms (S1 Fig and images of Lesser Antillean anoles in the appendices of [10]). Although the Bonaire site means of the hue traits (achromatic, green, blue dorsal) lie within the prediction intervals, they do not lie directly on the regression line. For example, a high achromatic dorsal value is reached before the xeric conditions are as extreme as on Bonaire (e.g., *A*. *aeneus* and *A*. *roquet*, Fig 2A) and *A*. *bonairensis* cannot be more than 100% achromatic due to the way hue is measured (proportional RGB). Intensity of green and other hues would be better measured by spectrometry and quantified as in [17], but this data was not available for all the comparative Lesser Antillean species.

For the scalation traits there was not enough species to employ site means. The secondary analysis using individuals (S3 File) suggests the low number of ventral scales along the body in the Bonaire anole is closely predicted by the southern Lesser Antillean xeric-montane comparison, but the number of dorsal scales gave no significant regression in the LA data available. Hence, what information is available on scalation indicates that, unlike the hue and pattern, the intraspecific variation is less consistently parallel and only equivocal capacity to predict interspecific variation. Natural selection experiments have shown scale number to be the targets of selection [12], and should respond to directional selection. The intraspecific variation in scale number of lizards in relation to climate has been discussed over a protracted period [10, 18, 30–32] with some generalizations pointing in opposing directions. Most of these discussions have focussed on the idea that increase in scale number equates to an increase in scale size and a possible increase in scale sculpturing with an impact of the surface area to volume relationship). However, more detailed and comprehensive study [10] show that the situation is quite complex, with different species (and even the same species among regions of Dominica) capable of trends in opposite directions. Different workers recording traits in different ways is obviously a general problem with this type of study and specifically with dorsal scalation data this can recorded either along the length trunk, or around the circumference of the trunk. This may to some extent explain the discrepancy among studies. Indeed scale number may not just reflect (inverse) scale size as such, but reflect body shape, with lizards with long thin gracile trunks having more dorsal scale along the body and fewer around the circumference, while lizards with short robust trunks have the opposite, irrespective of scale size. Hence, scale number may respond to a number of competing pressures (size re water loss, trunk shape) which may render prediction difficult, and/or unreliable.

With the hue and pattern there is a high degree of predictability of interspecific character states from intraspecific trends in congeners. In this specific sense, the evolution of the Bonaire anole appears largely deterministic rather than chaotic. There is much about anole evolution that appears deterministic, from the independent evolution of complex anole communities on the Greater Antilles [8], to the parallel evolution of xeric versus montane forms within solitary Lesser Antillean anoles [10]. The general question of the extent to which microevolution can predict macroevolution cannot be answered by the single study of a specific group, although these can contribute to a consensus. In any event, the arguments need to be evidence-based rather than assertive [2], and in this case the evidence suggests that the interspecific divergence of *A*. *bonairensis* is largely driven by the same natural selection pressures as the interspecific divergence within the anoles from the mountainous Lesser Antilles.

## Supporting information

S1 Fig Images of male specimens. Top, *A*. *bonairensis* from extremely xeric Bonaire showing achromatic dorsum and intense chevrons: Middle, Its closest phylogenetic relative in the Lesser Antilles, *A*. *Luciae* from the St Lucian rainforest, with intense green dorsum and no chevrons: Bottom, *A*. *Luciae* from the xeric habitat in St Lucia showing achromatic dorsum and slight chevrons. (NB individuals vary).
(TIF)

S1 Table Regression statistics. (DOCX)

S2 Table ANOVA table. ANOVAs for quantitative traits for *A*. *boniarensis* from 3 sites.
(PDF)

S1 File Climate data. (XLSX)

S2 File Quantitative trait data. (XLSX)

S3 File Scalation. (DOCX)
