# Supplementary figures and images for: Predictability in evolution: Adaptation of the Bonaire anole (Anolis bonairensis) to an extreme environment
Source: PLoS One. 2017 May 1;12(5):e0176434. doi: 10.1371/journal.pone.0176434 (PMC5411080; doi:10.1371/journal.pone.0176434)

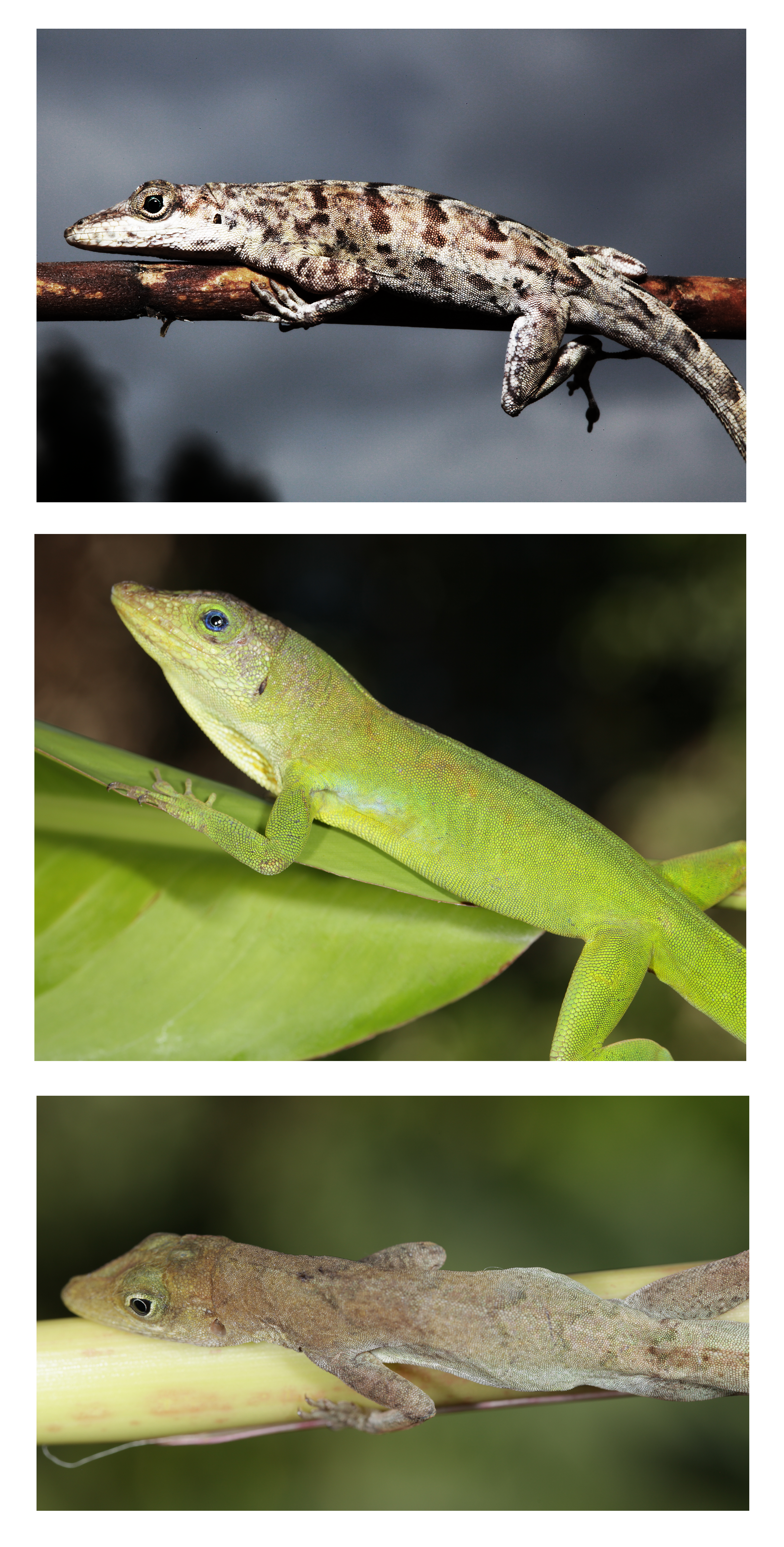

Supplement: S1 Fig — Top, A. bonairensis from extremely xeric Bonaire showing achromatic dorsum and intense chevrons: Middle, Its closest phylogenetic relative in the Lesser Antilles, A. Luciae from the St Lucian rainforest, with intense green dorsum and no chevrons: Bottom, A. Luciae from the xeric habitat in St Lucia showing achromatic dorsum and slight chevrons. (NB individuals vary). (TIF) [file pone.0176434.s001.tif]
