# Supplementary material for: Predictability in evolution: Adaptation of the Bonaire anole (Anolis bonairensis) to an extreme environment
Source: PLoS One. 2017 May 1;12(5):e0176434. doi: 10.1371/journal.pone.0176434 (PMC5411080; doi:10.1371/journal.pone.0176434)
Supplement: S1 Table — (DOCX) [file pone.0176434.s002.docx]

**Regression slopes and 95% confidence limits**

| **Trait** | **b** | **upper** | **lower** |  | **b_P_** | **upper** | **lower** |
| --- | --- | --- | --- | --- | --- | --- | --- |
| Achromatic dorsum | -13.15 | -8.16 | -18.13 |  | 11.87 | -8.20 | -15.56 |
| Green dorsum | 5.64 | 3.43 | 7.86 |  | 5.45 | 6.97 | 3.93 |
| Blue dorsum | -8.15 | -4.20 | -12.13 |  | -6.15 | -4.28 | -8.01 |
| Chevron intensity | -0.51 | -0.30 | -0.71 |  | -0.57 | -0.40 | -0.73 |

Legend. Regression slope b (pooled irrespective of species) and pooled within-group b_P_(within-species) slope (Snedecor & Cochran 1967) with their upper and lower 95% confidence limits.

GW Snedecor, WG Cochran 1989. Statistical Methods, Sixth Edition. The Iowa State University Press, Iowa, USA.
