# Supplementary material for: Predictability in evolution: Adaptation of the Bonaire anole (Anolis bonairensis) to an extreme environment
Source: PLoS One. 2017 May 1;12(5):e0176434. doi: 10.1371/journal.pone.0176434 (PMC5411080; doi:10.1371/journal.pone.0176434)
Supplement: S2 Table — ANOVAs for quantitative traits for A. boniarensis from 3 sites. (PDF) [file pone.0176434.s003.pdf]

ANOVAs

Trait	DFs	F	P
Achromaticity	2,19	2.9	0.08
Green	2,19	0.6	0.53
Blue	2,19	2.8	0.09
Chevron	-	-	-
Ventrals	2,19	3.6	0.05
Dorsals	2,19	0.4	0.66

ANOVAs for quantitative traits for *A. bonairensis* from 3 sites
