# Supplementary material for: Predictability in evolution: Adaptation of the Bonaire anole (Anolis bonairensis) to an extreme environment
Source: PLoS One. 2017 May 1;12(5):e0176434. doi: 10.1371/journal.pone.0176434 (PMC5411080; doi:10.1371/journal.pone.0176434)
Supplement: S3 File — (DOCX) [file pone.0176434.s006.docx]

**Scalation.**

An insufficient number of species met the criteria for inclusion for the scalation traits (ventral and dorsal scales). Consequently, the sample sizes were too low for site means to be employed and these traits were excluded from this set of analyses. Scalation has been the focus of attention in lizard, including anole, studies so these traits are analysed in this supporting information for exploratory purposes, using individuals instead of site means. All other procedures are as for the hue and pattern traits, for example, the regression lines are computed in the same way excluding the Bonaire site means.

There is no significant correlation between dorsal scale number and climate using the individuals from selected LA species (Table below) and this trait is excluded from further analysis.

| **Trait** | **N^a^** | **r^b^** | **p^c^** | **a^d^** | **b^e^** | **ucl** | **lcl** | **b_P_** | **ucl** | **lcl** |
| --- | --- | --- | --- | --- | --- | --- | --- | --- | --- | --- |
| Ventral scales**^1^** | 156 | 0.36 | <0.001 | 75.24 | 3.57 | 5.05 | 2.09 | 5.27 | 4.23 | 6.30 |
| Dorsal scales**^2^** | 61 | 0.14 | >0.05 | 82.22 | 2.39 | - | - | - | - | - |

**Table legend. ^a^** Sample size. **^b^** Correlation of QT and climate. **^c, d, e^** null hypothesis probability, intercept and slope of regression of QT against climate. Regression slope b, and pooled within-group (within-species) slope b_P_ with their 95% upper (ucl) and lower (lcl) confidence limits. **^1^** Individual values from *A. richardii* (Grenada) and *A.roquet* from northwest and central Martinique. **^2^** Individual values from *A. richardii* and *A. aeneus* (Grenada) .

There is a significant positive correlation/regression between the number of ventral scales and climate in some LA anoles. The Bonaire anole has few ventral scales as predicted by the situation in xeric LA anoles, with the means falling between the prediction intervals (Fig below). See Discussion in the main text.

40

60

80

100

-5

-3

-1

1

3

climate

ventrals

**Fig Legend. Regression plots for ventral scales against climate.** Horizontal axis pc1representing climate, with xeric low scores and montane rainforest high scores, vertical axis is number ventral scales along trunk. Regression slope (straight line) with upper and lower prediction interval curves for scatter points. Data points represent individual specimens except Bonaire which are site means. Species symbols (xeric red, montane green), *A. roquet* NW Martinique upright triangle, *A. roquet* Central Martinique inverted triangle, *A. richardii* Grenada four point star, and *A. bonairensis* Bonaire circle.
